# Dr. Arnold C. Hawes

**Published:** 1895-07

**Authors:** 


### Obituary.

### DR. ARNOLD C. HAWES.

On the 7th day of April last, Dr. Arnold C. Hawes, one of the earliest and most esteemed members of the New York Odontological Society, entered into his final rest.

Soon after the formation of the Society he was elected an active member, and so continued until he retired from practice and took up his residence at his country-seat in Connecticut. He then tendered his resignation and was made an honorary member.

Dr. Hawes was one of the founders of the First District Dental Society: its first vice-president and second president. He was one of the original members of the Dental Society of the State of New York, and for many years its treasurer. He was among the first members of the American Dental Association, and for a time served as treasurer of that organization.

Dr. Hawes was one of the most prominent and best respected members of our specialty: a man of sterling integrity, with generous impulses and candid manners. He was much interested in the work of our professional associations, and especially so in this, his favored society. He was also an occasional contributor of articles for our dental journals.

Most of the gentlemen present will remember his ever-genial, smiling face, and the warm grasp of his friendly hand. He was a model of good nature, winning the respect and love of all who knew him. You will, perhaps, also remember that for many months Dr. Hawes was almost totally blind, yet he bore his misfortune with a remarkable degree of cheerfulness; and when by a surgical operation his sight was partly restored, his joy and gratitude seemed unbounded.

In view of the departure of our late friend and fellow-member, it is fitting that we record our high appreciation of his excellent character as a gentleman and professional worker. Therefore be it

*Resolved*, That while we mourn the loss of one who for so long a time was our intimate associate, and who by his cheerful spirit and kindly disposition endeared himself to his friends and fellow-members, we are devoutly thankful that it was our privilege to enjoy his genial companionship and to have been classed among his friends. And in our affectionate remembrance comes also the cheering thought that he led a good and useful life, and earned an honorable record.

CHARLES E. FRANCIS,
C. A. WOODWARD,
ALBERT H. BROCKWAY,
*Committee.*

